# Bone morphogenic protein signaling in spinal cord injury

**DOI:** 10.20517/2347-8659.2020.34

**Published:** 2021-03-21

**Authors:** Nadia Al-Sammarraie, Swapan K. Ray

**Affiliations:** Department of Pathology, Microbiology, and Immunology, University of South Carolina School of Medicine, Columbia, SC 29209, USA

**Keywords:** Spinal cord injury, bone morphogenic protein, apoptosis, proliferation, autophagy, differentiation, inflammation

## Abstract

Spinal cord injury (SCI) is a debilitating injury that results from traumatic or non-traumatic insults to the spinal cord, causing significant impairment of the patient’s activity and quality of life. Bone morphogenic proteins (BMPs) are a group of polyfunctional cytokines belonging to the transforming growth factor beta superfamily that regulates a wide variety of cellular functions in healthy and disease states. Recent studies suggest that dysregulation of BMP signaling is involved in neuronal demyelination and death after traumatic SCI. The focus of this article is to describe our current understanding of the role of BMP signaling in the regulation of cell fate, proliferation, apoptosis, autophagy, and inflammation in traumatic SCI. First, we will describe the expression of BMPs and pattern of BMP signaling before and after traumatic SCI in rodent models and *in vitro*. Next, we will discuss the role of BMP in the regulation of neuronal and glial cell differentiation, survival, functional recovery from traumatic SCI, and the gap in knowledge in this area that requires further investigation to improve SCI prognosis.

## INTRODUCTION

Spinal cord injury (SCI) can be either traumatic or non-traumatic damage to the spinal cord and has a peak prevalence of approximately 906-1800 cases per million people in the United States^[1–3]^. SCI usually causes complete or partial motor and sensory neurological deficits with deleterious outcomes^[4]^. Traumatic SCI can be caused by major trauma to the spinal cord following road traffic accidents, falls in the elderly, and violent and sport-related injuries^[1,3]^. Non-traumatic SCI usually result from ischemic-reperfusion injury, congenital malformations, degenerative diseases, malignancy, autoimmune diseases, or infections in the spinal cord^[2,5,6]^. Traumatic SCI is a devastating neurological condition characterized by both acute and chronic phases of progressive spinal cord damage that involve neuroinflammation, oligodendrocytes loss, neuronal loss, demyelination, and reactive astrogliosis with scar formation^[7,8]^. The acute phase is characterized by oligodendrocyte death and demyelination, reactive astrocyte proliferation, axonal swelling, and acute inflammatory cell infiltration^[9,10]^. The chronic phase is characterized by chronic infiltration of inflammatory cells, partial neuronal regrowth and remyelination, and glial scar establishment^[10,11]^. The upregulation of detrimental factors and pathways cause progressive pathogenesis leading to the activation of cysteine proteases (calpains and caspases) for neuronal and glial cell death, and declining neurological function (motor and sensory) in both acute and chronic traumatic SCI^[12,13]^. Current research is mostly focused on traumatic SCI for understanding of its pathogenesis and developing effective new therapeutic strategies. The main goals in developing new therapies for traumatic SCI are to minimize neural cell loss and prevent glial scar formation to promote remyelination and functional recovery^[8,14]^.

**Figure 1.**
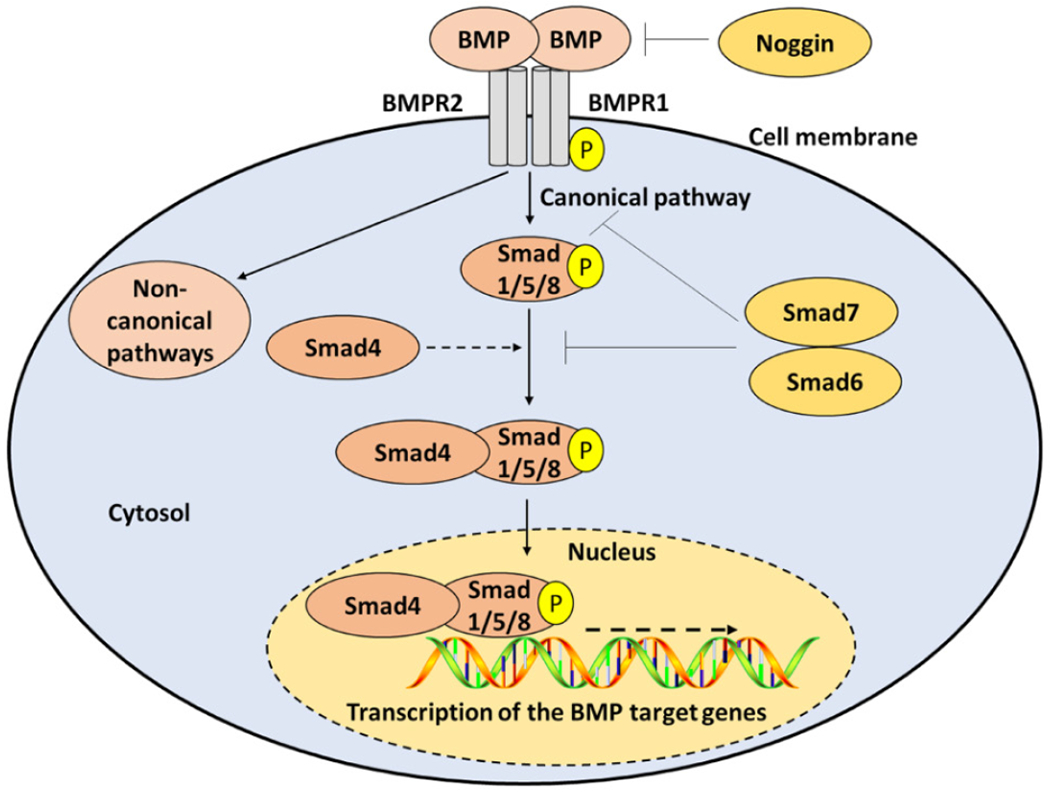
Molecular components and pathways of BMP signaling. The BMP signaling is initiated by the binding of BMP ligands to BMPR1 and BMPR2. In the canonical pathway, BMP receptors phosphorylate Smad1/5/8, which can bind to Co-Smad4 and are translocated to the nucleus to regulate the expression of target genes. In the non-canonical pathways, BMP receptors activate non-Smad pathways. Termination of BMP signaling is achieved by noggin, Smad6, and/or Smad7. BMP: bone morphogenic protein; BMPR: BMP receptor

Bone morphogenetic proteins (BMPs) are a group of approximately 15 growth regulating polyfunctional cytokines that belong to the transforming growth factor beta (TGFβ) superfamily and are widely expressed in both the intact and injured spinal cord^[15,16]^. Signal activation and transduction include the binding of BMP cytokines to BMP receptor 1 (BMPR1A, BMPR1B, or ActR-1A) and BMP receptor 2 complex, followed by phosphorylation and activation of Smad1/5/8 intracellular receptor regulated proteins or R-Smads^[15,17,18]^. Smad1/5/8 proteins then bind to the common Smad4 or the Co-Smad4 to form a complex, which is translocated to the nucleus to regulate transcription of BMP-targeted genes in a context dependent manner^[15,17,18]^. Inhibition of signaling is usually achieved via the activation and competitive binding of Smad6, Smad7, and noggin inhibitory proteins. Smad6 and Smad7 inhibit the interaction between BMP receptors and R-Smads and/or the interaction between the R-Smads and the common Smad4^[19,21]^. Noggin inhibitory proteins bind with high affinity to BMP ligand proteins and prevent their association with their receptors^[22]^ [Figure 1].

Growing evidence from rodent models of SCI^[16,23]^ show that BMP ligands and receptors are expressed in the intact spinal cord and are drastically upregulated post-injury. This is summarized in Table 1.

**Figure 2.**
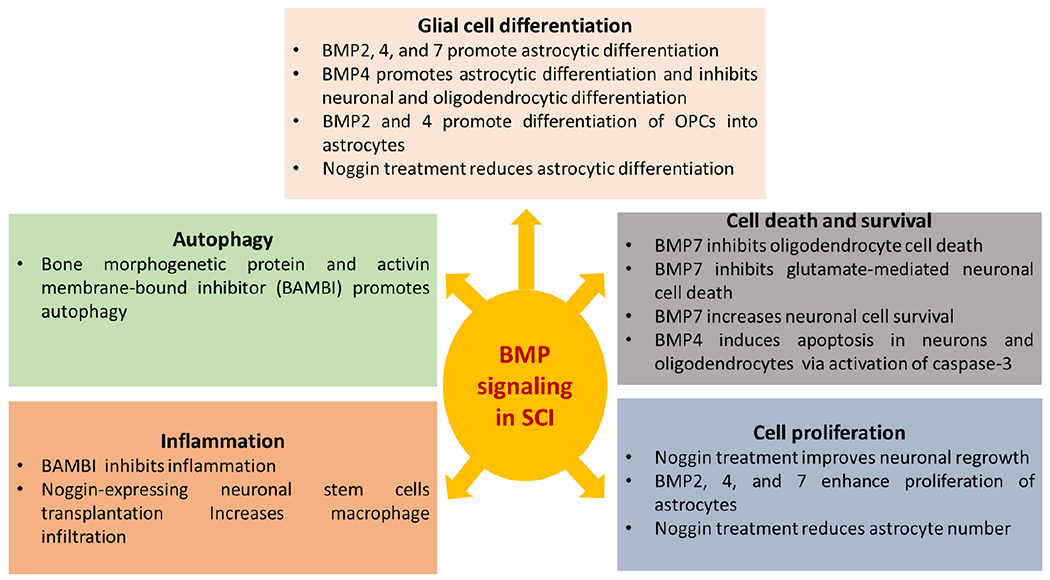
Cellular manifestations of BMP signaling in SCI. This diagram illustrates the *in vitro* and *in vivo* effects of activation or inhibition of BMP signaling on neuronal and/or glial cell proliferation, differentiation, survival, apoptosis, autophagy, and inflammation in SCI. BMP: bone morphogenic protein; SCI: spinal cord injury; BAMBI: BMP and activin membrane-bound inhibitor; OPCs: oligodendrocyte precursor cells

Furthermore, *in vitro* studies^[24,25]^ extensively elucidated the protective and deregulatory role of BMP components in a variety of cellular events on both neuronal and non-neuronal cells, which are summarized in Table 2. Most of these studies focused mainly on the level of expression of BMP signaling proteins and the resultant cellular damage, describing only limited knowledge on molecular regulation and downstream targets. This article will focus mainly on the role of different BMP ligands and receptors on neuronal and glial cell differentiation, neuroinflammation, cell death, and autophagy in the *in vivo* and *in vitro* models of traumatic SCI, which are summarized in Figure 2. Next, we will discuss the gap in knowledge in this area and suggest future studies for further understanding of the role of BMP signaling in pathogenesis in traumatic SCI that remains largely elusive.

## EXPRESSION OF BMP LIGANDS, RECEPTORS, AND SMAD AND NON-SMAD SIGNALING IN SPINAL CORD BEFORE AND AFTER INJURY, AND THEIR ASSOCIATION WITH FUNCTIONAL RECOVERY IN SCI

Studies in rodent models of SCI have shown that BMP ligands and receptors are expressed in intact spinal cord and their expression are further increased following SCI. BMP2, 3, 4, 5, 7, 9, 12, and 13 and the BMP receptors 1A, 1B, and 2 are minimally expressed in uninjured spinal cord^[16,23,26]^. However, after SCI, the expression of BMP ligands and their receptors are increased considerably in neuronal and glial cells in spinal cord and exert diverse cellular effects^[23,27]^. The increase in BMP2, 4, and 7 expression levels are amongst the most studied BMP ligands after SCI. Expression levels of the ligands and the downstream canonical pathway and non-canonical pathways were markedly increased in response to SCI. Chen *et al*.^[28]^ studied the expression of BMP4 and other signaling molecules critical for neuronal development in SCI in mice. This study found that BMP4 was upregulated after SCI in the neurons of the gray and white matter and ependymal cells (a type of glial cells known to produce cerebrospinal fluid and act as reservoir of neurodegeneration) surrounding the SCI lesion^[28]^. Setoguchi *et al*.^[27]^ studied the expression of BMP7 before and after acute SCI in rat model^[27]^. This study found that BMP7 was expressed in glial cells at low levels before injury but its expression was markedly increased in glial cells and expression occurred in motoneurons after SCI^[27]^ [Table 1].

Cui *et al*.^[29]^ conducted a study to examine the changes in expression of BMP2 and 4 in a rat model of SCI. This study found increases in expression of both BMP2 and 4 after SCI, which correlated with low Basso, Beattie, and Bresnahan (BBB) motor assessment scores when compared to controls^[29]^. Furthermore, they found that inhibition of BMP signaling using noggin treatment was able to improve BBB scores when compared to the untreated group after hemisection SCI^[29]^. Matsuura *et al.*^[23]^ studied the changes in expression of levels of BMP2 and 4 and the BMP receptor 2 in rats after SCI, and the effect of noggin treatment on recovery from SCI. They found that BMP2 and 4 and the BMP receptor 2 were slightly expressed in intact spinal cord and expression levels were further increased after SCI. Moreover, noggin treatment was able to improve locomotive function after SCI when compared to the non-treated SCI group^[23]^. Besides, several treatments with endogenous BMP components or recombinant BMP protein resulted in neuroprotective effects and improved locomotive function by modulating BMP signaling^[30,32]^. Kim *et al.*^[30]^ compared the effects of agmatine, an endogenous protein with neuroprotective effects, on scar formation and functional recovery after SCI in mice. This study found that agmatine reduced scar size and improved BBB scores, in part, by increasing expression of BMP7^[30]^. Park *et al*.^[31]^ showed that intraperitoneal agmatine treatment in a mice model of SCI was associated with increased expression of BMP2 and 7 in neurons and oligodendrocytes while expression of BMP4 in astrocytes and oligodendrocytes surrounding the damage site was reduced. The treatment resulted in improvement of locomotive function, inhibited neuronal death, and reduced scar size^[31]^. Similarly, Dmitriev *et al*.^[32]^ studied the effect of intra-thecal administration of rhBMP2 on expression of p-Smad1, 5, and 8 within the cells of the spinal cord after SCI in rats. The study found significant activation of p-Smad1, 5, and 8 in all neuronal cells, glial cells, and fibroblasts, which might affect recovery from SCI following rhBMP2 treatment [Table 3].

## ROLE OF BMP SIGNALING IN DIFFERENTIATION OF GLIAL CELLS AFTER SCI

Astrocytes, oligodendrocytes, ependymal cells, and microglia are non-neuronal heterogenous cell types that maintain spinal cord integrity, homeostasis, and myelination^[33]^. Marked increase in astrocyte differentiation was observed in response to SCI, which contributed to glial scar formation in SCI tissue^[34]^. On one hand, glial scar provides protective mechanisms to limit the lesion size after SCI; on the other hand, it leads to deleterious effects by the inhibition of axonal regeneration^[34,35]^. Recent studies suggest that BMP signaling promotes differentiation of neuronal stem cells (NSCs) and oligodendrocyte precursor cells (OPCs) into astrocytes predominantly^[36–38]^. Wang et al.^[36]^ studied the effect of the microenvironment created by reactive astrocytes on the differentiation of OPCs after SCI in rats. They found that SCI increased the expression of BMP4 in astrocytes isolated from the site of injury, and it further released BMP4 in their conditioning media. They also found that *in vitro* culture of OPCs in astrocytes-derived conditioning media activated Smad1, 5, and 8, which led to differentiation of a significant number of OPCs into astrocytes, while inhibiting differentiation of oligodendrocytes^[36]^.

In contrast, noggin treatment reduced astrocytic differentiation and increased oligodendrocytic differentiation^[36]^. Similarly, Xiao *et al*.^[37]^ conducted a study to test the effect of BMP signaling on the differentiation of NSCs after SCI in mice. This study found that BMP2, 4, and 7 were expressed in intact spinal cord and their expression was further increased after SCI in the following cell types: neurons, NSCs, microglia, and oligodendrocytes, but not in astrocytes^[37]^. They also found that the expression of phosphorylated Smad1, 5, and 8 were increased after SCI in the above cell types, OPCs, and astrocytes^[37]^. Furthermore, they found that BMP4 was highly expressed in neutrospheres (free-floating clusters of neural stem cells) cultured from the spinal cord and it promoted astrocytic differentiation from NSCs, while inhibition of BMP signaling using noggin treatment reduced astrocytic differentiation^[37]^. Setoguchi *et al*.^[38]^ examined the effect of BMPs on the differentiation of transplanted NPCs *in vitro* and after SCI in mice. This study found that BMP2 was expressed in the spinal cord before injury and was upregulated drastically after^[38]^. They also found that BMP2 and 7 promoted the differentiation of NPCs to astrocytes *in vitro*, while the inhibition of BMP signaling using Smad6, Smad7, or noggin overexpressing NPCs resulted in the differentiation of NPCs into neuronal cells and inhibited the differentiation of NPCs into astrocytes^[38]^. Similarly, transplanting the above-modified NPCs into a mice model of SCI resulted in improvement of the motor scores with inhibition of astrocytic differentiation and promotion of neuronal and oligodendrocytic differentiations *in vivo*^[38]^. Together, these studies imply that targeting BMP signaling could be beneficial for ameliorating astrocytic scar formation, and for enhancing oligodendrocytic differentiation for remyelination after SCI.

In addition, North *et al*.^[39]^ showed that the conditional deletion of β1 integrin from ependymal stem cells resulted in an increase in their differentiation into astrocytes, which could promote glial scar formation after SCI and reduce BBB motor scores in SCI mice, which were found to be associated with increases in canonical (Smad1/5/8) and non-canonical (p38) signaling. Furthermore, Song *et al*.^[25]^ found that BMP4 treatment of NSCs in culture promoted astrocytic differentiation via activation of Smad1/5/8, while noggin treatment resulted in reduction of astrocytic differentiation and an increase in oligodendrocytic differentiation *in vitro*. In contrast, Enzmann *et al*.^[16]^ showed that the intra-thecal transplantation of noggin overexpressing NSCs or progenitor cells was unable to restrict astrocytic differentiation in rats after SCI, suggesting additional regulatory mechanisms were controlling astrocytic differentiation.

Studies also showed that the expression of BMP receptors was increased after SCI, particularly affecting astrocytic hypertrophy (an astrocyte grown bigger than its normal size to adapt to changes) and differentiation. Astrocytes play both physiological and pathological roles after traumatic SCI, which triggers an initial astroctytic hypertrophy and subsequently, an astrocytic hyperplasia. In astrocytic hypertrophy, astrocytes are reactive with bigger bodies, thicker processes, and overexpression of their intermediate filament proteins such as glial fibrillary acidic protein and vimentin to help repair the blood-brain barrier and reduce the spread of inflammatory cells at the site of SCI. On the other hand, in astrocytic hyperplasia, astrocytes increase their numbers around the injury site and produce much finer processes to contribute to the development of the glial scar that becomes an impediment to axonal regeneration after SCI. Conditional deletion of astrocytic BMPR1A in mice has adverse effects on recovery after SCI via impairing astrocytic hypertrophy, reducing axonal density, and fostering inflammation^[26]^. In contrast, conditional deletion of astrocytic BMPR1B has more beneficial effects by increasing the number of hypertrophied astrocytes, attenuation of the glial scar, and diminishing lesion size in mice after SCI^[26]^. In addition, expression of BMPR1A, 1B, and 2 in OPCs that were transplanted into rat spinal cord predominantly promoted their differentiation into astrocytes^[40]^. Similarly, culture of OPCs in the presence or absence of BMP2/4 and noggin showed that BMP treatment increased their differentiation into astrocytes while noggin treatment enhanced it^[40]^.

## ROLE OF BMP SIGNALING IN AXONAL GROWTH AND GLIAL CELL PROLIFERATIONS AFTER SCI

Reactive astrogliosis (also known simply as astrogliosis or astrocytosis) and neuronal regrowth occur in response to the loss of glial cells and neurons after SCI to partially promote healing of tissue damage and attempt neuronal recovery^[10,41]^. Recent studies suggest that BMP signaling causes astrocytic proliferation and neuronal growth after SCI^[23,37,42]^. Parikh *et al*.^[42]^ studied whether Smad1 activation could have a beneficial effect on axonal regeneration in mice after SCI. BMP4 overexpression in dorsal motoneurons was achieved by the intra-thecal administration of viral vectors overexpressing BMP4 in mice after SCI^[42]^. The results show activation of Smad1 in dorsal neurons, which is associated with improving axonal growth after SCI^[42]^. The administration of recombinant mouse noggin intra-thecally improved locomotive function and increased axonal regrowth after SCI^[23]^. Xiao *et al*.^[37]^ found that the levels of BMP2, 4, and 7 expression were all increased in neurons, microglia, oligodendrocytes, and NSCs after SCI, which enhanced astrocytic proliferation, while noggin treatment diminished astrocyte numbers.

## ROLE OF BMP SIGNALING IN AUTOPHAGY AFTER SCI

Autophagy or “self-eating” is a central molecular mechanism that regulates tissue homeostasis in health and disease^[43]^. Autophagy is characterized by direct or indirect lysosomal degradation of damaged mitochondria, misfolded proteins, and other cellular debris for recycling to maintain energy metabolism in response to stressful stimuli^[43]^. Macroautophagy is the major type of autophagy, which includes sequential events of autophagosome formation, autophagosome-lysosome fusion, and autolysosomal degradation of cargos^[44]^. Autophagy flux is defined as the total dynamics of autophagy and thereby it is the progression of cargo sequestration into autophagosomes, delivery to lysosomes, and degradation by lysosomal enzymes^[45]^. Autophagy flux is usually increased in mechanical injury such as mild traumatic SCI or metabolic stress such as starvation, but autophagy flux is decreased due to suppression of autophagy at an upstream (autophagosome formation) or downstream step (autolysosome formation)^[46]^. Recent studies suggest an impairment of autophagy flux after moderate to severe SCI, which leads to neuronal cell death and adversely affects oligodendrocyte-mediated neuronal myelination and functional recovery^[47,48]^. On the other hand, activating autophagy improves neurological recovery in rodent models of SCI due to activation of autophagosome formation and/or enhancement of autophagy flux^[49,51]^. Although modulation of autophagy plays a crucial role in the pathogenesis in SCI, there is limited knowledge on the role of BMP signaling in the regulation of autophagy after SCI. BMP and activin membrane-bound inhibitor (BAMBI) is a pseudo-receptor that lacks the kinase activity and inhibits the signaling of TGFβ family^[52]^. BAMBI has been found to be down regulated in rats after SCI, while intraspinal injection of the BAMBI expressing vector after SCI promotes autophagy and improves locomotive function in rats^[52]^. The BAMBI overexpression causes activation of Beclin-1 and LC3B II, two proteins critical for inducing autophagy and maintaining autophagy flux; on the other hand, it results in down regulation of autophagy inhibitor proteins such as Bim and p62^[52]^. The role of BMP ligands and receptors in disruption of autophagy post-SCI remains largely unknown; however, modulating BMP signaling to restore autophagy may provide a new therapeutic avenue in treating SCI.

## ROLE OF BMP SIGNALING IN INFLAMMATION AFTER SCI

Inflammation is encountered in both the acute and chronic phases of SCI and results in expansion of the initial lesion, destruction of nearby tissue, neuronal loss, axonal demyelination, and fibrosis or scar formation^[53]^. Shortly after SCI, there is massive infiltration of neutrophils and after 24 hours, infiltration of reactive microglia/macrophages increases progressively^[9]^. Several weeks following SCI, there is an increase in the infiltration of CD45-positive cells and CD68-positive reactive microglia/macrophages, which mark the phase of chronic inflammation in SCI and are associated with impairment of locomotive function^[11]^. Recent studies suggest contradictory roles of the inhibition of BMP signaling on inflammatory responses after SCI. The transplantation of noggin-expressing NSCs in SCI rats results in a marked increase in macrophage infiltration^[16]^. In contrast, the overexpression of BAMBI in a rat model of SCI results in inhibition of neuroinflammation, which is characterized by reduction in the levels of expression of interleukin (IL)-1β, IL-6, IL-10, TGFβ, and mechanistic target of rapamycin^[54]^.

## ROLE OF BMP SIGNALING IN NEURONAL AND GLIAL CELL DEATH AFTER SCI

Both neurons and oligodendrocytes are highly susceptible to cellular damage and death following SCI resulting in axonal demyelination and neurological deficits^[7,55]^. *In vitro* and *in vivo* studies have shown that BMP7 exerts beneficial effects on neuronal and oligodendrocyte cell survival^[24,56]^ . de Rivero Vaccari *et al*.^[24]^ studied the protective mechanism of BMP7 on neuronal survival after SCI *in vitro* and *in vivo*. BMP7 promotes neuronal survival after SCI in rats and inhibits glutamate induced neuronal cell death *in vitro*^[24]^. Similarly, Wang *et al*.^[56]^ tested the effects of BMP7 on the survival of oligodendrocytes *in vitro*. Results showed that BMP7 treatment prevented tumor necrosis factor α-induced oligodendrocyte death. These results imply that BMP7 protects neurons and oligodendrocytes from cell death in SCI models. On the other hand, a recent study conducted by Hart *et al*.^[57]^ has shown that BMP4 induces apoptosis in both neurons and oligodendrocytes via the activation of caspase-3 (the final executioner of apoptosis) after SCI, while its inhibition using BMP signaling inhibitors attenuates the activation of caspase-3. These results suggest different roles of different BMP ligands on neuronal and glial cell survival post-SCI, which requires further investigation.

## CONCLUSION AND FUTURE PROSPECTIVE

BMP ligands, receptors, and inhibitors are differentially expressed in the intact spinal cord in rodents. BMP ligands, receptors, and canonical and non-canonical pathways are upregulated after SCI. In general, augmented BMP signaling results in adverse cellular responses and impairs functional recovery in SCI animal models. On the other hand, the inhibition of BMP signaling improves neuronal cell survival, neuronal outgrowth, and functional recovery after SCI. Although BMP dysregulation is reported in SCI, the cell-type specific role of BMP signaling in SCI remains poorly understood. Several gaps in knowledge still exist regarding the molecular mechanisms underlying BMP dysregulation, the direct causal link between individual BMP ligands and receptors and progression of pathogenesis in SCI, the spatial and temporal effects of BMP signaling in the pathogenesis of acute, subacute, and chronic phases in SCI, and the mechanisms by which BMP ligands regulate autophagy, inflammation, differentiation, and apoptosis. Further *in vivo* studies using conditional knockout rodent models are needed to understand the specific requirements of different BMP ligands in SCI and neurological recovery, the ligand-receptor pairs that are involved in the regulation of SCI pathogenesis, and the downstream canonical or non-canonical pathways that impact neuronal survival after SCI.

## Figures and Tables

**Table 1.** Expression of BMP signaling components before and after SCI in rodent models

BMP signaling component	SCI model	Outcomes	Ref.
BMP ligands	Rats	BMP7 mRNA was mildly expressed in glial cells in intact spinal cord but markedly expressed in glial cells and motoneurons post-SCI	[27]
	Rats	BMP2/4 mRNA was mildly expressed in intact spinal cord but markedly expressed in oligodendrocytes, astrocytes, and microglia surrounding the damaged site post-SCI	[23]
	Mice	BMP2, 4, and 7 levels were increased in neurons, microglia, oligodendrocytes, and NSCs post-SCI, which enhanced astrocyte proliferation. BMP4 promoted differentiation of astrocytes and inhibited differentiation of neurons and oligodendrocytes	[37]
	Rats	BMP2 and 4 levels were increased post-SCI and promoted differentiation of the engrafted OPCs cells into astrocytes	[40]
	Mice	BMP7 expression was increased after SCI and further augmented after agmatine treatment, leading to reduced collagen scar formation and improved BBB score post-SCI	[30]
	Rats	BMP4 expression was increased in astrocytes cultured from injured thoracic spinal cord	[36]
	Rats	BMP2/4 expression was increased after SCI and associated with low BBB scores	[29]
	Rats	BMP7 was expressed in glial cells of the intact spinal cord and increased in glial cells and motoneurons after SCI	[27]
	Mice	BMP2 was slightly expressed in intact spinal cord and markedly increased post-SCI	[38]
	Mice	BMP4 level was increased in neurons of gray and white matter and ependyma cells near the damaged site post-SCI	[28]
	Rats	BMP4 was overexpressed after acute SCI	[57]
	Rats	BMP2, 3, 4, 5, 7, 9,12, and 13 were expressed in intact spinal cord	[16]
BMP receptors	Rats	BMPR1A and BMPR2 expression levels were increased in neurons post-SCI	[23]
BMP antagonists	Rats	Noggin was minimally expressed in intact spinal cord	[16]
Canonical pathway	Mice	p-Smad1, 5, and 8 were activated in neurons, oligodendrocytes, OPCs, astrocytes, and NSCs post-SCI	[37]

BMP: bone morphogenic protein; SCI: spinal cord injury; OPCs: oligodendrocyte precursor cells; NSCs: neural stem cells; BBB: Basso, Beattie, and Bresnahan; BMPR: BMP receptor

**Table 2.** Effects of BMP treatment on neuronal and non-neuronal cells in SCI in *in vitro* models

BMP signaling component	Treatment	Outcomes	Ref.
BMP ligands	BMP7	BMP7 inhibited tumor necrosis factor α-mediated oligodendrocyte death	[56]
	BMP7	BMP7 inhibited glutamate induced neuronal cell death	[24]
	BMP4	*In vitro* culture of NSCs in the presence of BMP4 resulted in amelioration of oligodendrocyte differentiation and increase in astrocyte differentiation. Smad1 and 5 were activated in response to BMP4 treatment of NSCs	[25]
	BMP7	Noggin expressing OPCs treated with BMP7 showed less astrocytic differentiation	[16]
BMP antagonists	Noggin	Noggin treatment reduced astrocyte numbers. Inhibition of BMP4 using noggin attenuated differentiation of NSCs into astrocytes	[37]
	Noggin	Noggin treatment of OPCs partially reduced astrocytic differentiation	[40]
	Noggin	Noggin treatment reduced differentiation of OPCs into astrocytes in astrocyte conditioning media. p-Smad1, 5, and 8 levels were increased in OPCs in astrocyte conditioning media compared to control. OPCs cultured in astrocyte conditioning media predominantly differentiated into astrocytes	[36]
	Noggin and LDN193189	Treatment attenuated BMP4 induced activation of caspase-3 for cell death in neurons and oligodendrocytes post-SCI	[57]
	Noggin	Noggin treatment reduced astrocytic differentiation and increased the differentiation of NSCs into oligodendrocytes	[25]

BMP: bone morphogenic protein; SCI: spinal cord injury; OPCs: oligodendrocyte precursor cells; NSCs: neural stem cells

**Table 3.** Therapeutic and genetic targeting of BMP signaling in SCI in *in vivo* models

Treatment	SCI model	Effects	Ref.
BMP7	Rats	BMP7 promoted neuroprotection via an increase in the number of surviving neurons, in part, via increased p38 non-canonical signaling	[24]
Agmatine	Mice	It augmented BMP7 expression, reduced collagen scar formation, and improved BBB scores	[30]
Agmatine	Mice	It reduced neuronal cell death and scar formation, leading to improved locomotive function. This effect was achieved, in part, via increased expression of BMP2/7 in neurons and oligodendrocytes, and decreased expression of BMP4 in the damaged site	[31]
Conditional deletion of astrocytic BMPR1A and 1B	Mice	Knockouts of astrocytic BMPR1A cause reduction in astrocytic hypertrophy, decrease in axonal density, and enhancement of the inflammatory response. In contrast, knockouts of astrocytic BMPR1B increase astrocytic hypertrophy and reduce lesion size and glial scar formation post-SCI	[26]
Transplantation of OPCs expressing BMPR1A, 1B, and 2	Rats	Transplantation of OPCs expressing (BMPR1A, 1B, and 2) into rat spinal cord led to their differentiation into astrocytes	[40]
Administration of AAV vector encoding BMP4	Mice	Intra-thecal administration of AAV vector encoding BMP4 led to Smad1 activation in dorsal motoneuron and increased axonal regrowth after SCI	[42]
Conditional knockout of β1-integrin in ependymal stem cells	Mice	Conditional knockout of β1-integrin in ependymal stem cells increased the movement of BMPR1B into lipid rafts while enhancing BMP signaling (canonical and non-canonical) and glial scar formation	[39]
Noggin	Rats	Administration of recombinant mouse noggin intra-thecally improved locomotive function post-SCI and enhanced axonal regrowth	[23]
Noggin	Rats	Noggin treatment reduced BMP2/4 expression and improved motor scored post-SCI	[29]
Transplantation of Smad6, Smad7, or noggin expressing NPCs	Mice	It promoted differentiation of NPCs into oligodendrocytes and neurons but inhibited their differentiation into astrocytes, leading to improvement of BBB scores in mice post-SCI	[38]
Transplantation of noggin expressing neuronal stem Cells	Rats	It led to macrophage infiltration and widening of lesion size, but prevented astrocytic differentiation post-SCI	[16]
BAMBI	Rats	Overexpression of BAMBI inhibited inflammation and promoted autophagy post-SCI	[54]
BMP2	Rats	Intra-thecal administration of rhBMP2 resulted in increases in expression of p-Smad1, 5, and 8 in most spinal cord cell types	[32]

BMP: bone morphogenic protein; SCI: spinal cord injury; OPCs: oligodendrocyte precursor cells; NSCs: neural stem cells; BBB: Basso, Beattie, and Bresnahan; BMPR: BMP receptor; BAMBI: BMP and activin membrane-bound inhibitor; AAV: adeno-associated virus
